# Intracorporeal vs extracorporeal anastomosis in laparoscopic right colectomy for colon cancer: a prospective multicenter cohort study (the Hemi-D-TREND study)

**DOI:** 10.1007/s00464-025-12401-0

**Published:** 2025-12-01

**Authors:** Xavier Serra-Aracil, Mireia Pascua-Solé, Antonio Sánchez, Carlos-Javier Gómez-Díaz, Cristina Ruiz, Beatriz Espina, José Enrique Sierra, Susana Lamas, Helena Vallverdú, Constanza Corredera, Carlos Veo, Carlos Hoyuela, Anna Serracant, Felix Moreno, Pablo Collera-Ormazabal, María-José Mañas, Mireia Merichal, Ladislao Cayetano-Paniagua, Aleidis Caro-Tarragó

**Affiliations:** 1 Coloproctology Unit, Department of Surgery, General and Digestive Surgery Service, Parc Tauli University Hospital, Universitat Autònoma de Barcelona, Institut d’investigació I Innovació Parc Tauli I3PT-CERCA, Parc Tauli s/n, 08208 Sabadell, Barcelona, Spain; 2 Coloproctology Unit, General and Digestive Surgery Department, Consorci Hospitalari de Terrassa, Terrassa, Barcelona, Spain; 3 Coloproctology Unit, General and Digestive Surgery Department, Hospital Universitari Sant Joan de Reus, Tarragona, Spain; 4 ALTHAIA, Xarxa Assistencial Universitària de Manresa—Sant Joan de Déu Hospital, Manresa, Spain; 5 Coloproctology Unit, General and Digestive Surgery Department, Hospital Santa Tecla de Tarragona, Tarragona, Spain; 6 Coloproctology Unit, General and Digestive Surgery Department, Hospital Universitari Joan XXIII de Tarragona, Tarragona, Spain; 7 Coloproctology Unit, General and Digestive Surgery Department, Hospital Universitari Arnau de Vilanova de Lleida, Lleida, Spain; 8 Coloproctology Unit, General and Digestive Surgery Department, Hospital Universitari de Vic, Barcelona, Spain; 9 Coloproctology Unit, General and Digestive Surgery Department, Hospital de Sant Boi, Barcelona, Spain; 10 Coloproctology Unit, General and Digestive Surgery Department, Hospital de Cancer Barretos, Barretos, Brazil; 11 Coloproctology Unit, General and Digestive Surgery Department, Hospital de Mollet, Barcelona, Spain

**Keywords:** Right colectomy, Intracorporeal anastomosis, Extracorporeal anastomosis, Anastomotic leak, Laparoscopy, Enhanced recovery

## Abstract

**Background:**

Anastomotic leak (AL) is the most severe complication after laparoscopic right colectomy (RC), with historical median rates around 8%. Whether intracorporeal ileocolic anastomosis (ICA) offers advantages over extracorporeal anastomosis (ECA) under standardized, purely laparoscopic conditions remains uncertain. We aimed to compare AL rates and short-term postoperative outcomes between ICA and ECA in laparoscopic RC for colon cancer.

**Methods:**

Prospective multicenter cohort (TREND-compliant) across 11 hospitals (January 2019–June 2022). Adults with non-metastatic right colon cancer undergoing elective laparoscopic RC were included. Exposure (ICA vs ECA) was determined by each hospital’s routine practice. Primary outcome: AL, per predefined clinical, radiologic, or endoscopic criteria. Secondary outcomes: conversion to open surgery, length of stay (LOS), complications (Clavien–Dindo), surgical site infection (SSI), and a composite of severe complications (COSC). Analyses used the full cohort; propensity score matching (PSM) was prespecified as a sensitivity analysis.

**Results:**

A total of 438 patients were analyzed: 225 ICA and 213 ECA. AL occurred in 3/225 (1.33%) after ICA and 3/213 (1.41%) after ECA (*p* = 1.00; risk difference − 0.08 percentage points; 95% CI − 2.1 to 2.3). Conversion was lower with ICA (2.2% vs 7.5%; *p* = 0.013), while LOS was shorter with ICA (median 4 days; *p* < 0.001). There were no significant differences in severe morbidity (Clavien–Dindo ≥ III: 5.8% ICA vs 3.8% ECA; *p* = 0.375), SSI (incisional or organ/space), COSC (6.7% ICA vs 4.2% ECA; *p* = 0.298), reoperation, or mortality. Findings were consistent in PSM analyses (213:213).

**Conclusions:**

In this prospective multicenter laparoscopic cohort, both intracorporeal and extracorporeal anastomosis achieved anastomotic-leak rates below 2%, with no superiority of one technique over the other regarding leak or severe morbidity. ICA was associated with lower conversion and shorter hospital stay. These results confirm the overall safety and feasibility of both approaches in experienced centers.

**ClinicalTrials.gov Identifier:**

NCT03918369.

Anastomotic leak (AL) after colorectal resection is the most serious postoperative complication due to its impact on morbidity, mortality, hospital stay, and the frequent need for an ostomy [1–3]. Until a decade ago, there were no data from large series on AL rates or associated risk factors. The ANACO group’s multicenter study of 3193 patients across 52 hospitals reported an AL rate of 8.7% [3].

The importance of ileocolic AL after right colectomy (RC) has often been underestimated. In a 2015 European Society of Coloproctology study involving 3208 patients, the AL rate was 8.1% [4]. A subanalysis by the ANACO group in 1102 RC patients found an AL rate of 8.4% (range 0–35%) [5], attributed to variations in patient profiles, surgical technique, and AL definitions [6].

Laparoscopic side-to-side intracorporeal anastomosis (ICA) has emerged as a standardized, reproducible option. Reported AL rates around 2% [7–9], also seen in robotic surgery, support its use [10]. Its main limitations are technical complexity and longer operative time.

Some studies link ICA to reduced morbidity and fewer surgical site infections. However, recent meta-analyses have not conclusively demonstrated superiority over extracorporeal anastomosis (ECA) [11, 12]. Most findings suggest ICA is at least non-inferior in terms of complications and recovery.

The study was prospectively designed and is reported in accordance with the TREND (Transparent Reporting of Evaluations with Nonrandomized Designs) guidelines. In addition, our reporting aligns with key elements of the TARGET guidance for observational studies emulating a target trial, as we frame a parallel-group comparison of ICA versus ECA in routine practice with prespecified eligibility, time zero (day of surgery), outcomes, and baseline-confounder adjustment.

We hypothesized that laparoscopic RC with ICA (RC-ICA group) would yield better outcomes than with ECA (RC-ECA group), particularly regarding AL, morbidity, and mortality. The primary objective of this study was to compare AL rates between both techniques in a multicenter, purely laparoscopic setting, applying standardized procedures and strict selection criteria while excluding robotic surgery. Secondary objectives included conversion to open surgery, postoperative length of stay, 30-day morbidity (Clavien–Dindo), reoperation, and 30-day mortality.

## Material and methods

### Study design

This was a prospective, multicenter, controlled cohort study with a non-randomized design, conducted in accordance with the TREND (Transparent Reporting of Evaluations with Non-randomized Designs) guidelines [13, 14]. It compared laparoscopic right colectomy (RC) with mechanical intracorporeal side-to-side anastomosis (RC-ICA group) versus RC with extracorporeal anastomosis (RC-ECA group).

The study protocol and consent forms were approved by the institutional review boards of all participating centers. The Ethics Committee of Parc Taulí University Hospital acted as the reference (ID: HemiD-TREND 2018/658). The study was registered at ClinicalTrials.gov (ID: NCT03918369) and conducted in accordance with the Declaration of Helsinki and the TREND non-randomized study guidelines [13, 14]. The study protocol has been previously published by our team [15].

### Patients: patient selection

Consecutive patients with right colon neoplasia confirmed by colonoscopy and CT scan were eligible. Surgeons were members of coloproctology units performing > 30 laparoscopic RCs per year using ICA or ECA as standard practice. Eligible patients provided written informed consent.

Inclusion criteria: Adults (≥ 18 years) with non-metastatic right colon cancer and patients with benign right-colon lesions deemed at high risk of malignancy scheduled for elective oncologic laparoscopic RC. The right colon was defined preoperatively on CT as including the terminal ileum, cecum, ascending colon, and the hepatic flexure. The transverse colon beyond the hepatic flexure was not considered part of the right colon in this study [5]. No patients were excluded postoperatively due to discrepancies between preoperative imaging and intraoperative findings. Patients had to have followed the perioperative management program in place at each hospital.

Exclusion criteria: Non-right colon cancer, open or emergency surgery, stage cT4 on preoperative CT scan (as determined by the institutional multidisciplinary colorectal cancer board) or IV of the TNM classification [16], ASA (American Society of Anesthesiologists) IV, serum albumin ≤ 3.4 g/dL, BMI < 18 or > 35 kg/m^2^, pregnancy, cirrhosis, dialysis, non-standard ICA technique [17, 18]; or failure to provide informed consent.

### Intervention

Patients underwent either RC-ICA or RC-ECA. Both procedures began with medial-to-lateral dissection.

RC-ICA: Only standardized intracorporeal anastomoses were accepted, defined as a side-to-side isoperistaltic anastomosis performed with a linear stapler (Endopath® Echelon Flex™ 60). The enterotomy was closed with a continuous suture, with or without reinforcement using Monocryl™ (poliglecaprone 25) or STRATAFIX™ Spiral knotless suture. The specimen was extracted through a Pfannenstiel incision.

RC-ECA: Both manual and mechanical techniques (side-to-side or end-to-side) were accepted, given their widespread standardization and the lower variability in outcomes reported in the literature [15]. The specimen extraction site was chosen by the surgeon.

### Study variables

#### Primary outcome for both RC-ICA and RC-ECA groups

Anastomotic leak, defined by Peel et al. [19], is the leakage of luminal content from a surgical join between two hollow viscera, diagnosed (1) radiologically, by enema with water-soluble contrast or by CT with the presence of intra-abdominal collections adjacent to the anastomosis; (2) clinically, with evidence of extravasation of intestinal content or gas through a wound or drain; (3) by endoscopy; or (4) intraoperatively.

#### Secondary outcomes

Demographic and preoperative variables: hospital, age, sex. Additional preoperative variables: classification, body mass index (BMI).

Surgical variables: surgical time, type of anastomosis (manual/mechanical); type of anastomosis (side-to-side or end-to-side); size in cm and location of the minilaparotomy; blood loss, surgery performed by a staff surgeon or resident; scores of risk prediction models for evaluating the homogeneity of the groups using POSSUM [20] and CR-POSSUM [21].

Postoperative variables: Conversion to open surgery (defined as the need to perform a midline laparotomy or to enlarge the minilaparotomy beyond 10 cm) [22]; postoperative pain assessment according to the visual analog scale (VAS) on days 1 and 2 post-surgery. Variables at 30 days post-surgery: overall morbidity, morbidity according to the Cl-D classification [23], relevant morbidity (Cl-D > II), Comprehensive Complication Index score (CCI) [24], surgical site infection (SSI), as defined by the Center for Disease Control [25] in its subdivisions of incisional and organ-space SSI; AL requiring surgical treatment; composite variable of severe complication (COSC) [10]; nosocomial infection; surgical complications (postsurgical bleeding, incisional SSI, organ-space SSI and AL); medical complications; surgical reintervention; mortality; hospital stay and pathology variables (T/N).

### Allocation method

The surgical technique (ICA or ECA) was determined by the institutional standard of each participating hospital. Six centers routinely performed intracorporeal anastomosis (ICA), while five centers routinely performed extracorporeal anastomosis (ECA). Within each hospital, all eligible patients underwent the same standardized technique, independent of surgeon preference or patient characteristics, thereby reducing the risk of intra-institutional selection bias. Although some surgeons were technically capable of performing both approaches, for the purposes of this study they adhered strictly to the institutional protocol. No preoperative clinical or tumor-related criteria were used to assign patients to one technique over the other. Thus, allocation was defined at the hospital level rather than at the individual level, minimizing intra-hospital bias but precluding patient-level randomization.

### Sample size

The sample size calculation was based on an expected anastomotic leak (AL) rate of 8% for RC-ECA [4, 5] and 2% for RC-ICA [7]. With a two-sided α = 0.05 and 80% power, a minimum of 208 patients per group was required to detect this 6-percentage point absolute difference. Allowing for a 10% attrition rate, the total required sample size was 416 patients.

### Masking

Data were collected prospectively and centrally by the sponsoring center in a secure online database created by the firm Xolomon (copyright©2013 XolomonTree S.L). All participants had access only to their own patients and had no knowledge of the results of the other hospitals. Only the principal investigator knew the results of the entire study. For greater data reliability, 30% of the patients were randomly monitored in person by the Academic Research Organization platform at the Vall d’Hebron Hospital Research Institute in Barcelona.

### Statistical analysis

Variable description and statistical analysis were performed using the Statistical Package for the Social Sciences (SPSS Inc., Chicago, IL) version 26, along with R and R Studio. Prospective data collection, centralized management, and external monitoring ensured a minimal rate of missing data (< 1%), which were excluded from the analyses.

Quantitative variables were expressed as means with standard deviations or medians with interquartile ranges, depending on distribution. Categorical variables were presented as counts and percentages. Normality was assessed using the Kolmogorov–Smirnov test. Comparisons between independent groups were performed using Student’s *t*-test or the Mann–Whitney *U* test for quantitative variables, and Pearson’s Chi-square or Fisher’s exact test for categorical variables, as appropriate. A *p*-value < 0.05 was considered statistically significant, and 95% confidence intervals were reported when relevant.

To control for potential confounding and selection bias related to treatment allocation by center, a 1:1 Propensity Score Matching (PSM) was performed using the matchit package in R. Two matching methods were tested: nearest-neighbor matching and optimal matching, both without replacement. The propensity score was estimated using a logistic regression model including baseline covariates such as age, sex, BMI, and ASA score. Post-matching balance between groups was assessed using standardized mean differences, and only matched pairs were retained for further analysis.

In both the matched sample and the full cohort, binary logistic regression was applied to identify factors associated with the composite outcome of severe complication (COSC). Variables with *p* < 0.1 in univariate analysis were included in the multivariable model. Multicollinearity was assessed prior to model construction.

Because allocation was defined at the hospital level, only certified colorectal units with homogeneous perioperative pathways and standardized use of a single anastomotic technique were included. Given the limited number of centers (*n* = 6) and the intentional homogeneity among sites, no hierarchical or GEE model was applied, as between-center variability was minimal and largely inherent to the exposure definition.

## Results

### Patient flow and recruitment

**Fig. 1:**
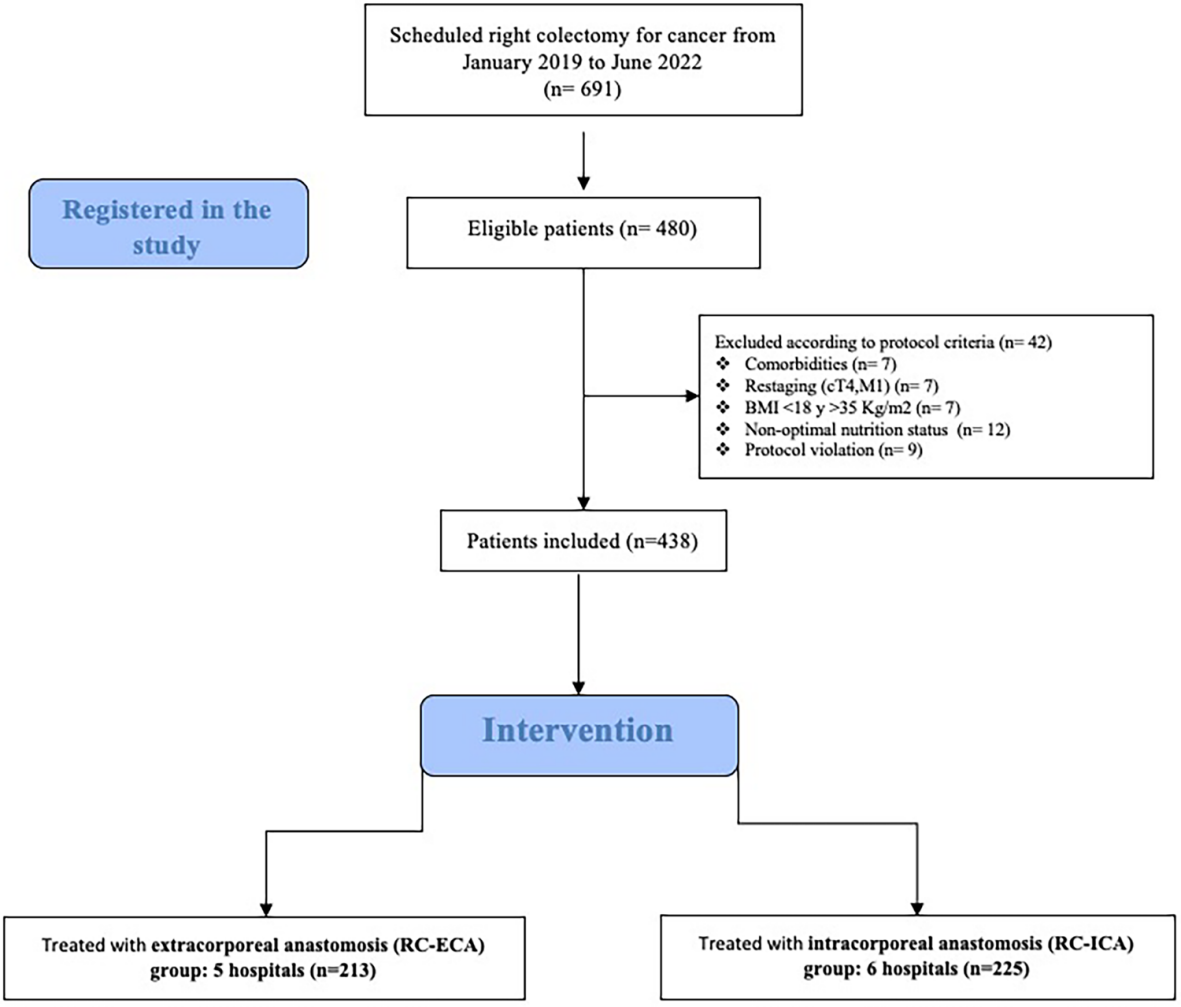
Flowchart of eligibility and center-level treatment strategy (ECA vs ICA)

Between January 2019 and June 2022, 691 patients diagnosed with right colon neoplasia underwent RC at 11 hospitals (Fig. 1). Of these, 480 (69.5%) met the initial selection criteria and were entered into the database. Ultimately, 438 patients were included in the analysis: 225 in the RC-ICA group and 213 in the RC-ECA group. The prospective design and centralized real-time data entry ensured minimal missing data, allowing complete-case analysis without the need for imputation.

### Primary outcome

Anastomotic leak (AL) occurred in 6/438 patients (1.37%) overall: 3/225 in the RC-ICA group (1.33%) and 3/213 in the RC-ECA group (1.41%). There were no statistically significant differences between groups (*p* = 1.00; risk difference − 0.08 percentage points; 95% CI − 2.1 to 2.3).

### Secondary outcomes

#### Demographic and preoperative variables

Mechanical bowel preparation, prehabilitation, and ERAS adherence were higher in the RC-ICA group (Table 1).

**Table 1:** Demographic, preoperative variables

Characteristics	Total (*n* = 438)	RC-ECA group (*n* = 213)	RC-ICA group (*n* = 225)	*p* value difference (ICA-ECA), 95% CI
Demographic and preoperative variables				
Sex (*n*, %)				
Male	183 (41.8)	96 (45.1)	87 (38,7)	0.18^b^
				6.4 (15.6 to − 2.8)
Female	255 (58.2)	117 (54.9)	138 (61.3)	
Age median (IQR) (years)	72 (14)	72 (14)	72 (16)	.536
Preoperative tests				
ASA (*n*, %)				
ASA I	16 (3.7)	6 (2.8)	10 (4.4)	0.31^a^
ASA II	243 (55.5)	113 (53.1)	130 (57.8)	
ASA III	179 (40.9)	94 (44.1)	85 (37.8)	
BMI, median (IQR) (kg/m^2^)	26.7 (5.9)	26.8 (5.8)	26.6 (6.2)	0.76
Colon mechanical preparation (*n*, %)	338 (77.2)	133 (62.4)	205 (91.1	< 0.001^b^
				28.3 (21.2 to − 36.2)
Prehabilitation (*n*, %)	186 (42.5)	56 (26.3)	130 (57.8)	< 0.001^b^
				31.5 (22.7 to 40.2
ERAS (*n*, %)	321 (73.3)	126 (59.2)	195 (86.7)	< 0.001^b^
				27.5 (19.6 to − 35.5)

*RC-ECA* Right colectomy—Extracorporeal anastomosis, *RC-ICA* Right colectomy—Intracorporeal anastomosis, *ASA* American Society of Anesthesiologists, *BMI* Body mass index, *ERAS* Enhanced Recovery After Surgery, *IQR* Interquartile range, *95% CI* 95% confidence interval

^a^Pearson Chi-square

^b^Fisher’s exact test

#### Surgical variables

The RC-ICA group showed significantly longer operative time, (Table 2) greater use of mechanical and side-to-side anastomoses, smaller minilaparotomy, and more frequent suprapubic incisions. Only 28 patients (6.4%) were operated on by residents, with fewer in the RC-ICA group (3.6%).

**Table 2:** Surgical variables

Characteristics	Total (*n* = 438)	RC-ECA group (*n* = 213)	RC-ICA group (*n* = 225)	*p* value difference (ICA − ECA), 95% CI
Surgical time (IQR) (min)	150 (70)	110 (60)	175 (55)	< 0.001^b^
Manual/mechanical anastomosis (*n*, %)				
Mechanical	413 (94.3)	188 (88.3)	225 (100)	< 0.001^b^
Manual	25 (5.7)	25 (11.7)	0 (0)	− 11.7 (− 7.4 to − 16.1)
Type of reconstruction (*n*, %)				
Side-to-side	349 (79.7)	124 (58.2)	225 (100)	< 0.001^b^
End-to-side	89 (20.3)	89 (41.8)	0 (0)	− 41.8 (− 35.2 to− 48.4)
Reinforcement suture (*n*, %)	154 (35.2)	53 (24.9)	101 (44.9)	< 0.001^b^
				− 20 (− 11.3 to − 28.7)
Location of minilaparotomy				
Lumbar region	186 (42.5)	185 (86.9)	1 (0.4)	< 0.001^a^
Midline	33 (7.5)	28 (13.1)	5 (2.2)	
Suprapubic (Pfannenstiel)	219 (50)	0	219 (97.3)	
Length of minilaparotomy (IQR) (cm)	6 (2)	7 (2)	5 (1)	< 0.001
Intraop. blood loss, median (IQR) (range) (mL)	0 (0) (0–1000)	0 (0) (0–1000)	0 (0) (0–600)	0.69
Surgeon’s experience				
Resident	28 (6.4)	20 (9.4)	8 (3.6)	0.02^b^
Staff	410 (93.6)	193 (90.6)	217 (96.4)	5.8 (10.4 to1.2)
Predictive models of risk (POSSUM, CR-POSSUM)				
POSSUM Physiology Severity Score, median (IQR)	18 (6)	18 (7)	18 (6)	0.26
POSSUM Operative Severity Score, median (IQR)	10 (0)	10 (0)	10 (1)	0.16
POSSUM prediction of morbidity (%), median (IQR)	25 (20.5)	24.4 (19)	27 (21.2)	0.22
POSSUM prediction of mortality (%), median (IQR)	4.4 (4.2)	4.3 (3.7)	4.9 (4.2)	0.29
CR-POSSUM physiological condition score, median (IQR)	10 (3)	10 (3)	10 (2.5)	0.88
CR-POSSUM intervention severity score, median (IQR)	7 (0)	7 (0)	7 (0)	0.41
CR-POSSUM prediction of mortality (%), median (IQR)	2.6 (2.3)	2.6 (2.3)	2.6 (1.8)	0.77

*RC-ECA* Right colectomy—extracorporeal anastomosis, *RC-ICA* Right colectomy—Intracorporeal anastomosis, *IQR* Interquartile Range, *95% CI* 95% confidence interval, *Cl-D* Clavien-Dindo

^a^Pearson Chi-square

^b^Fisher’s exact test

The application of the POSSUM morbidity–mortality and CR-POSSUM mortality risk prediction models did not show statistically significant differences between the two groups.

#### Postoperative and pathology variables

Conversion to open surgery occurred in 21 patients (4.8%), (Table 3) significantly more frequent in RC-ECA (16/213 [7.5%]) than RC-ICA (5/225 [2.2%]), *p* = 0.013; 95% CI [–9.3 to –5.3]). In a post hoc analysis restricted to the ECA group (*n* = 213), Conversion to open surgery was not associated with manual versus mechanical construction (1/25 [4.0%] vs 15/188 [8.0%], *p* = 0.7), but was significantly more frequent with end-to-side (T–L) anastomosis than with side-to-side (L–L) (15/89 [16.9%] vs 1/124 [0.8%], *p* < 0.001).

**Table 3:** Postoperative and pathology assessment variables

Characteristics	Total (*n* = 438)	RC-ECA group (*n* = 213)	RC-ICA group (*n* = 225)	P value Difference (ICA − ECA), 95% CI
Conversion to open surgery	21 (4.8)	16 (7.5)	5 (2.2)	0.01^b^
				− 5.3 (− 1.3 to − 9.3)
VAS 1 st day post op. median (IQR)	2 (2)	2 (2)	2 (2)	0.32
VAS 2nd day post op. median (IQR)	1 (2)	1 (2)	1 (2)	0.382
Overall morbidity				
No	287 (65.5)	148 (69.5)	139 (61.8)	0.11^b^
Yes	151 (34.5)	65 (30.5)	86 (38.2)	− 7.7 (1.2 to − 16.6)
Clavien-Dindo (Cl-D)				
Cl-D 0	287 (65.5)	148 (69.5)	139 (61.8)	0.57^a^
Cl-D I	75 (17.1)	32 (15)	43 (19.1)	
Cl-D II	55 (12.6)	25 (11.7)	30 (13.3)	
Cl-D IIIa	3 (0.7)	1 (0.1)	2 (0.9)	
Cl-D IIIb	14 (3.2)	5 (2.3)	9 (4)	
Cl-D IVa	3 (0.7)	2 (0.9)	1 (0.4)	
Cl-D IVb	0	0	0	
Cl-D V	1 (0.2)	0	1 (0.4)	
Relevant Cl-D (Cl-D > II)				
Cl-D ≤ II	417 (95.2)	205 (96.2)	212 (94.2)	0.38^b^
Cl-D > II	21 (4.8)	8 (3.8)	13 (5.8)	− 2 (2 to − 6)
Comprehensive Complication Index: mean (SD)	0 (0.9)	0 (0.8)	0 (0.11)	0.896
SSI	13 (3)	4 (1.9)	9 (4)	0.19^b^
				− 2.1 (1 to 5.3)
Incisional SSI	7 (1.6)	3 (1.4)	4 (1.8)	1^b^
				− 0.8 (2 to 2.7)
Organ Space SSI	9 (2.1)	3 (1.4)	6 (2.7)	0.51^b^
				− 1.3 (1.4 to − 3.9)
Anastomotic leak	6 (1.4)	3 (1.4)	3 (1.3)	1^b^
				0.1 (− 2.1 to 2.3
AL requiring surgery (Clavien–Dindo ≥ IIIa)	5	2 (0.9)	3 (1.3)	1^b^
				− 0.4 (1.6 to − 2.4)
Composite Outcome Severe Complication (COSC)	24 (5.5)	9 (4.2)	15 (6.7)	0.3
				− 2.4 (1.8 to − 6.7
Nosocomial infection	11 (2.5)	7 (3.3)	4 (1.8)	0.37^b^
				1.5 (4.5 to − 1.4)
Surgical complications	79 (18)	29 (13.6)	50 (22.2)	0.03^b^
				− 8.6 (− 1.5 to − 15.7)
Postop bleeding	59 (13.5)	29 (13.6)	30 (13.4)	1^b^
				0.3 (6.7 to − 6.1)
Medical complications	48 (11)	18 (8.5)	30 (13.3)	0.16^a^
				0.3 (6.7 to − 6.1)
Repeat surgery	15 (3.4)	5 (2.3)	10 (4.4)	0.3^b^
				− 2.1 (1.3 to − 5.5)
Mortality	1 (0.2)	0 (0)	1 (0.4)	1^b^
				− 0.4 (0.4 to − 1.3)
Hospital stay (days), median (IQR)	4 (2)	4 (1)	4 (2)	< 0.001
Pathology study				
T stage				
T0	72 (16.4)	29 (13.6)	43 (19.1)	0.07^a^
T1	45 (10.3)	24 (11.3)	21 (9.3)	
T2	80 (18.3)	40 (18.8)	40 (17.8)	
T3	183 (41.8)	83 (39)	100 (44.4)	
T4	58 (13.2)	37 (17.4)	21 (9.3)	
Advanced vs initial T stage				
T0-1–2	197 (45)	93 (43.8)	104 (46.2)	0.63^b^
T3-4	241(55)	120 (56.2)	121 (53.8)	− 2.6 (6.8 to − 11.9)
N stage				
N0	320 (73.1)	156 (73.2)	164 (72.9)	0.76^a^
N1	81 (18.5)	41 (19.2)	40 (17.8)	
N2	37 (8.4)	16 (7.5)	21 (9.3)	

*RC-ECA* Right colectomy—extracorporeal anastomosis, *RC-ICA* Right colectomy—Intracorporeal anastomosis, *SD* standard deviation, *IQR* interquartile range, *95% CI* 95% confidence interval, *Cl-D* Clavien-Dindo, *SSI* surgical site infection

^a^Pearson Chi-square

^b^Fisher’s exact test

Hospital stay was significantly shorter in the RC-ICA group (*p* < 0.001). Postoperative complications (postsurgical bleeding, incisional SSI, organ/space SSI, AL, and incisional hernia) did not differ significantly between groups. The higher surgical-complication rate observed in 50 patients (22.2%) in the RC-ICA group and in 29 (13.6%) in the RC-ECA group (*p* = 0.025; 95% CI [–15.7 to –1.5]) was mainly due to minor events such as postoperative bleeding, wound or organ/space infections, and transient ileus (Clavien–Dindo ≤ II). No significant differences were found in severe complications (Clavien–Dindo > II), CCI, reoperation, or mortality.

### Ancillary analyses

Table 4 displays the demographic, preoperative, and surgical variables that comprise the COSC variable. Only VAS on the second day and reinforcing suture presented differences < 0.1. In the binary logistic regression analysis, VAS on the second day emerged as a predictor with an odds ratio of 1.6 (95% CI 1.3–2.1).

**Table 4:** Demographic, preoperative and surgical variables included in the Composite Outcome Severe Complication (COSC)

Characteristics	Total (*n* = 438)	Cl-D ≤ II-SSI (*n* = 414)	Cl-D > II + SSI (*n* = 24)	*p* value difference (95% CI)
Demographic and preoperative variables				
Sex (*n*, %)				
Female	183 (41.8)	175 (95.6)	8 (4.4)	0.524
Male	255 (5.2)	239 (93.7)	16 (6.3)	32.7 (50.1 to − 14.8)
Age, median (IQR) (years)	72 (14)	72 (15)	73.5 (12)	0.391
Preoperative tests				
ASA (*n*, %)				
ASA I	16 (3.7)	14 (87.5)	2 (12.5)	0.129
ASA II	243 (55.5)	234 (96.3)	9 (3.7)	
ASA III	179 (40.9)	166 (92.7)	13 (7.3)	
BMI, median (IQR) (kg/m^2^)	26.7 (5.9)	26.6 (5.85)	27.8 (6.1)	0.178
Mechanical bowel preparation (*n*, %)	338 (77.2)	321 (95)	17 (5)	0.453
				6.7 (25.3 to − 11.9)
Prehabilitation (*n*, %)	186 (42.5)	178 (95.7)	8 (4.3)	0.401
				9.7 (29.2 to − 9.8)
ERAS	321 (73.3)	305 (95)	16 (5)	0.479
				7 (26.3 to − 12.3)
Surgical and postoperative variables				
Surgical time (IQR) (min)	150 (70)	140 (70)	151.5 (40)	0.292
ECA/ICA (*n*, %)				
RC-ECA	213 (48.6)	204 (95.8)	9 (4.2)	0.3
RC-ICA	225 (51.4)	210 (93.3)	15 (6.7)	11.8 (8.2 to − 31.7
Manual/mechanical anastomosis (*n*, %)				
Mechanical	413 (94.3)	390 (94.4)	23 (5.6)	1
Manual	25 (5.7)	24 (96)	1 (4)	− 1.6 (6.7 to − 9.9)
Type of mechanical anastomosis (*n*, %)				
Side-to-side	349 (79.7)	329 (94.3)	20 (5.7)	0.798
End-to-side	89 (20.3)	85 (95.5)	4 (4.5)	− 3.9 (11.5 to − 19.3)
Reinforcement suture (*n*, %)	154 (35.2)	150 (97.4)	4 (2.6)	0.076
				19.6 (35.2 to 4)
Location of minilaparotomy				
Right lumbar area	186 (42.5)	179 (96.2)	7 (3,8)	0.326
Midline	33 (7.5)	30 (90.9)	3 (9.1)	
Pfannenstiel	219 (50)	205 (93.6)	14 (6.4)	
Length of minilaparotomy median (IQR) (cm)	6 (2)	6 (2)	5 (2.4)	0.119
Intraop blood loss. median (IQR)	0 (0)	0 (0)	0 (0)	0.19
Surgeon’s experience				
Resident	28 (6.4)	27 (96.4)	1 (3.6)	1
Staff	410 (93.6)	387 (94.4)	23 (5.6)	
Conversion to open surgery	21 (4.8)	19 (90.5)	2 (9.5)	0.322
				− 3.7 (7.5 to − 15)
VAS 1 st day post op. median (IQR)	2 (2)	2 (2)	2 (2)	0.829
VAS 2nd day post op. median (IQR)	1 (2)	1 (2)	2 (2)	< 0.001
T stage (advanced vs initial)				
T0-1–2	197 (45)	186 (94.9)	11 (5.1)	1
T3-4	241(55)	227 (94.5)	14 (5.5)	− 3.5 (16.8 to − 23.8)
N stage				
N0	320 (73.1)	304 (95)	16 (5)	0.701
N1	81 (18.5)	76 (93.8)	5 (6.2)	
N2	37 (8.4)	34 (91.9)	3 (8.1)	

*RC-ECA* Right colectomy—Extracorporeal anastomosis, *RC-ICA* Right colectomy—Intracorporeal anastomosis, *ASA* American Society of Anesthesiologists score, *BMI* Body mass index, *ERAS* Enhanced recovery after surgery, *IQR* Interquartile range. *95% CI* 95% confidence interval, *Cl-D* Clavien-Dindo, *SSI* surgical site infection

### Propensity score matching (PSM)

Propensity score matching (nearest-neighbor and optimal) yielded 213:213 matched pairs. Because baseline imbalances were already small, nearest-neighbor matching did not further reduce them, whereas the optimal algorithm achieved modest additional balance. In the matched cohorts, no between-group differences reached statistical significance for the evaluated outcomes; estimates were directionally similar with wider uncertainty.

## Discussion

This was a pragmatic, prospective multicenter cohort in which the anastomotic approach (ICA vs ECA) reflected each hospital’s routine practice. The study was designed and reported in accordance with the TREND (Transparent Reporting of Evaluations with Nonrandomized Designs) guidelines [13, 14]. To enhance clarity and transparency, our reporting also aligns with key principles of the recently proposed TARGET guidance for observational studies emulating a target trial [26]—explicitly specifying eligibility, time zero (day of surgery), treatment strategies (ICA vs ECA as center-level routine practice), outcomes, and prespecified adjustment for baseline confounders (including propensity score methods). Because exposure was determined at the center level, the design reduces within-hospital selection but may introduce between-hospital confounding.

Participating hospitals were experienced in both techniques, yet each routinely used a single approach. The review board deemed randomization between approaches unethical where one was not the local standard. As Victora et al. [27] have emphasized, randomized trials are not always practical or ethical for evaluating health interventions in real-world settings. Within these constraints, a nonrandomized, center-level exposure design offered the most feasible and transparent approach. To minimize potential between-hospital confounding, we included only certified colorectal units with comparable perioperative pathways and a single standardized anastomotic strategy in routine practice, and we adjusted remaining imbalances—including ERAS adherence, prehabilitation, and extraction-site/incision—using multivariable analyses and propensity-score matching.

Our primary endpoint showed no superiority between techniques: anastomotic leak occurred in 3/225 (1.33%) after ICA and 3/213 (1.41%) after ECA (*p* = 1.00), yielding AL < 2% in both groups. For secondary outcomes, ICA was associated with lower conversion to open surgery (2.2 vs 7.5%, *p* = 0.013) and shorter hospital stay (median 4 days, *p* < 0.001), while no differences were observed in severe morbidity, SSI, reoperation, or mortality. In propensity score–matched analyses (213:213), no between-group differences reached statistical significance, with directionally similar estimates and wider uncertainty.

To reduce variability, RC-ECA was limited to side-to-side or end-to-side techniques (manual or mechanical), and only laparoscopic approaches were included, excluding robotic surgery. This improves the external comparability of results.

The absence of randomization required the introduction of all possible compensatory mechanisms [26]: the use of selection criteria that excluded factors associated with AL in previous studies [3, 4, 28]; the application of predictive risk models such as POSSUM [20] and CR-POSSUM [21]; the use of an online database managed by an independent firm, the randomized monitoring of 30% of the study data by an independent firm; the logistic regression analysis of the COSC; and the use of PSM statistical analysis.

The ICA technique offers advantages, but these are offset by the need for considerable skill in laparoscopic surgery and the longer learning curve. In our study, the median operative time in the ICA group was 175 min, compared to 110 min in the ECA group, with statistically significant differences, as previously reported [11, 29].

High-quality prospective controlled data directly comparing RC-ICA versus RC-ECA are scarce [29, 30]. Recent meta-analyses are inconclusive regarding superiority, with pooled estimates generally favoring intracorporeal anastomosis or being compatible with noninferiority [31, 32]. Consistent with our findings, the multicenter MIRCAST study reported AL rates < 2% with no significant differences between ICA and ECA [10]. Our study adds value by focusing exclusively on laparoscopic right colectomy for cancer using standardized techniques in high-volume centers, enhancing applicability to routine laparoscopic practice.

Although our hypothesis of ICA superiority for AL was not confirmed, both techniques achieved very low AL rates (< 2%) under standardized, purely laparoscopic conditions [7–9]. The unexpectedly low rate observed with ECA contrasts with prior multicenter reports (~ 8%) [4, 5] and likely reflects the study context: specialized coloproctology units, consistent use of each center’s standard approach by experienced teams, prospective monitoring, and predefined eligibility that excluded known high-risk features for leak [7–9]. Despite lower uptake of ERAS and prehabilitation in ECA centers, these factors did not show a conclusive impact on postoperative complications in our analyses. Residual between-hospital confounding remains possible, but sensitivity analyses (including propensity-score matching) were consistent with the main findings.

These findings are consistent with contemporary prospective series—including MIRCAST [10]—reporting low ECA leak rates in expert, high-volume units (≈1–6%) [4, 5]. In our cohort, strict eligibility (well-nourished patients without locally advanced disease) and the exclusive involvement of specialized colorectal surgeons using a uniform laparoscopic technique likely contributed to < 2% AL in both groups. While this reflects current optimized practice, it also lowers the number of events and thus reduces the statistical power to detect small between-group differences, as acknowledged among the study’s limitations.

Although ICA theoretically offers advantages by avoiding exteriorization of the bowel ends and minimizing mesenteric traction and colonic manipulation—factors that may improve perfusion and reduce tissue trauma—our data do not demonstrate a lower AL rate compared with ECA. In experienced colorectal units, standardized ECA can achieve similar outcomes, supporting that surgical expertise and adherence to technical principles are more determinant than the anastomotic approach itself. Nevertheless, ICA remains technically demanding, requires advanced laparoscopic proficiency, and may prolong operative time, which should be acknowledged when interpreting its wider applicability.

Overall complications were higher in the RC-ICA group (38.2% vs. 30.5%), though not statistically significant and mostly Clavien-Dindo ≤ II. Surgical complications were more frequent with RC-ICA; however, rectal bleeding rates were similar. Despite shorter incisions in RC-ICA, VAS pain scores on postoperative days 1–2 were comparable. Despite the absence of differences in Relevant Cl-D (Cl-D > II) postoperative complications, hospital stay was shorter in the RC-ICA group, in agreement with other studies [32, 33]. As in most published studies, surgical time was longer in the RC-ICA group, although this did not impact the rest of the variables used to assess postoperative complications.

Infectious complications did not differ between groups. As in MIRCAST [10], the COSC composite (Table 4) showed no between-group differences [34]. In multivariable logistic regression, only VAS > 2 on postoperative day 2 predicted COSC (OR 1.6, 95% CI 1.3–2.1) [35]. Conversion occurred in 4.8%, comparable to other series [7, 10], and was significantly lower with RC-ICA despite greater procedural complexity, in contrast to some series reporting different conversion patterns [32, 33, 36]. In an exploratory post hoc analysis restricted to ECA, conversion appeared higher with end-to-side than side-to-side anastomosis (16.9 vs 0.8%; *p* < 0.001), with no signal for manual versus mechanical construction. As the study was not designed to test configuration-specific effects and this association is sparsely described in the literature, it should be interpreted cautiously.

Although differences in conversion rate and length of stay were statistically significant, their absolute magnitude was modest and should be interpreted cautiously. Nonetheless, in expert, high-volume centers, even small improvements in these parameters may offer meaningful efficiency and recovery benefits.

This study has several limitations. The allocation of ICA versus ECA was determined at the hospital level rather than randomized at the patient level. Although this approach minimized intra-institutional selection bias, it may have introduced inter-institutional confounding factors that should be considered when interpreting the results. The strict selection criteria, which led to the inclusion of 69.5% of all potentially eligible patients. This increases internal validity but partially limits external validity. In addition, in the RC-ECA group more than one type of anastomosis was occasionally performed. The recruitment period also coincided with the COVID-19 pandemic, which prolonged the study excessively. Resident participation was more frequent in the ECA group; however, this variable was included in both the multivariable and propensity-score models. It was not identified as an independent predictor of anastomotic leak or severe morbidity and was adequately balanced between groups after matching.

Finally, the < 2% anastomotic-leak rate observed in both groups—well below the ~ 8% assumed a priori from contemporary multicenter studies [4, 5]—substantially reduced the power to assess non-superiority for the primary endpoint. Moreover, the very small number of events (*n* = 6; 3 per group) precluded meaningful adjusted modeling for AL and widened confidence intervals. Consistent with the prospectively registered protocol and good research practice, the pre-specified sample size was not modified post hoc; therefore, estimates for the primary endpoint should be interpreted with caution.

Future studies planned within this project will focus on the relative impact of both procedures on oncologic outcomes, long-term cost-effectiveness—including functional and occupational recovery—and the incidence of incisional hernia. Finally, another limitation is the absence of data on smoking and alcohol consumption as potential risk factors for anastomotic leakage, which were not included in the original study design.

## Conclusions

Both ICA and ECA achieved very low anastomotic leak rates (< 2%) under standardized laparoscopic conditions. While ICA was associated with shorter length of stay and lower conversion, it was not superior to ECA in terms of leak or severe morbidity. These findings suggest that, in expert settings, both techniques represent safe and effective options, with ICA showing potential recovery benefits when performed by experienced laparoscopic surgeons, owing to its greater technical complexity.

## Data Availability

The study data are available in the federated and multidisciplinary data repository of Catalan universities, CERCA research centers and other research entities: CORA.RDR. This repository complies with the FAIR principles (ensure that data is findable, accessible, interoperable, and reusable) and follows the EOSC (European Open Science Cloud) guidelines. The dataset related to this study has been published in the dataverse (own space within the repository) of the Institut d’Investigació i Innovació Parc Taulí, the institution to which the first author of the publication is affiliated. The dataset is available at: 10.34810/data1722. The set of data deposited contains a database with all the clinical data from the study, a codebook that describes the variables of the study presented in the database file and a “readme” with all the descriptions, explanations, and relationships of files. These have been deposited under a CC BY-NC-SA 4.0 use and distribution license, that permits to share (copy and distribute the material in any medium or format) and adapt (remix, transform, and build upon the material) the data. In addition, you must give appropriate credit to the original authors (attribution), not use the material for commercial purposes, and if you remix, transform, or build upon the material, you must distribute your contributions under the same license as the original. Access and downloading of the files are completely free to guarantee open access to the data and facilitate its use in the scientific community, always under the terms of use and distribution mentioned above.
